# Model organism as boundary object: *Physarum polycephalum* between biology, network science and art

**DOI:** 10.1007/s40656-026-00770-1

**Published:** 2026-11-05

**Authors:** Maria Dębińska

**Affiliations:** Institute of Archaeology and Ethnology Polish Academy of Sciences, Warsaw, Poland

**Keywords:** *Physarum polycephalum*, Network models, Boundary object, Equivocation

## Abstract

The slime mold *Physarum polycephalum* is a single celled Protist known for creating efficient protoplasmic networks connecting its food sources. The properties of *Physarum* networks have been subject to extensive examination and employed to solve mathematical problems as well as imitate, model and optimize human-made networks. This paper investigates the conceptual operations underpinning those modelling practices that allow for comparison between two incommensurate entities: the slime mold versus human transportation and communication systems. It analyzes selected *Physarum* experiments, describes the process of abstraction of the slime mold’s body into networks and identifies the strategies of comparison between humans and the slime mold. It defines the slime mold as a boundary object performing the work of translation between the fields of biology, network science and art and characterizes the methods of arriving at equivalence between human and slime networks as based on analogy, metaphor and equivocation.

## Introduction

In the last two decades *Physarum polycephalum*, also known as The Blob (Dussutour & Bailly, 2022), has taken by storm the fields of biology, computer science and bio-art, due to a number of remarkable feats it has managed to accomplish in the field of network creation and optimisation. The aim of this paper is to characterize it as an atypical model organism and explore the various types of network modelling practices it affords, focusing on the methods of comparison between slime mold and human networks.

*Physarum*^1^ is a single-celled eukaryotic organism belonging to the phylum of Amoebozoa and the infraphylum of Mycetozoa, which gathers cellular and acellular slime molds. When nutrients are plentiful, slime molds live as solitary, single-celled amoebae that forage independently. When nutrients become scarce, they aggregate into a larger structure—either a multicellular slug or, as in the case of *Physarum polycephalum* and other members of the order Physarales, a plasmodium: a giant, multinucleate cell that can span tens of square centimetres and is visible to the naked eye. The plasmodial cell can divide into autonomous parts that forage separately but merge back together when encouraged by external conditions.

**Fig. 1:**
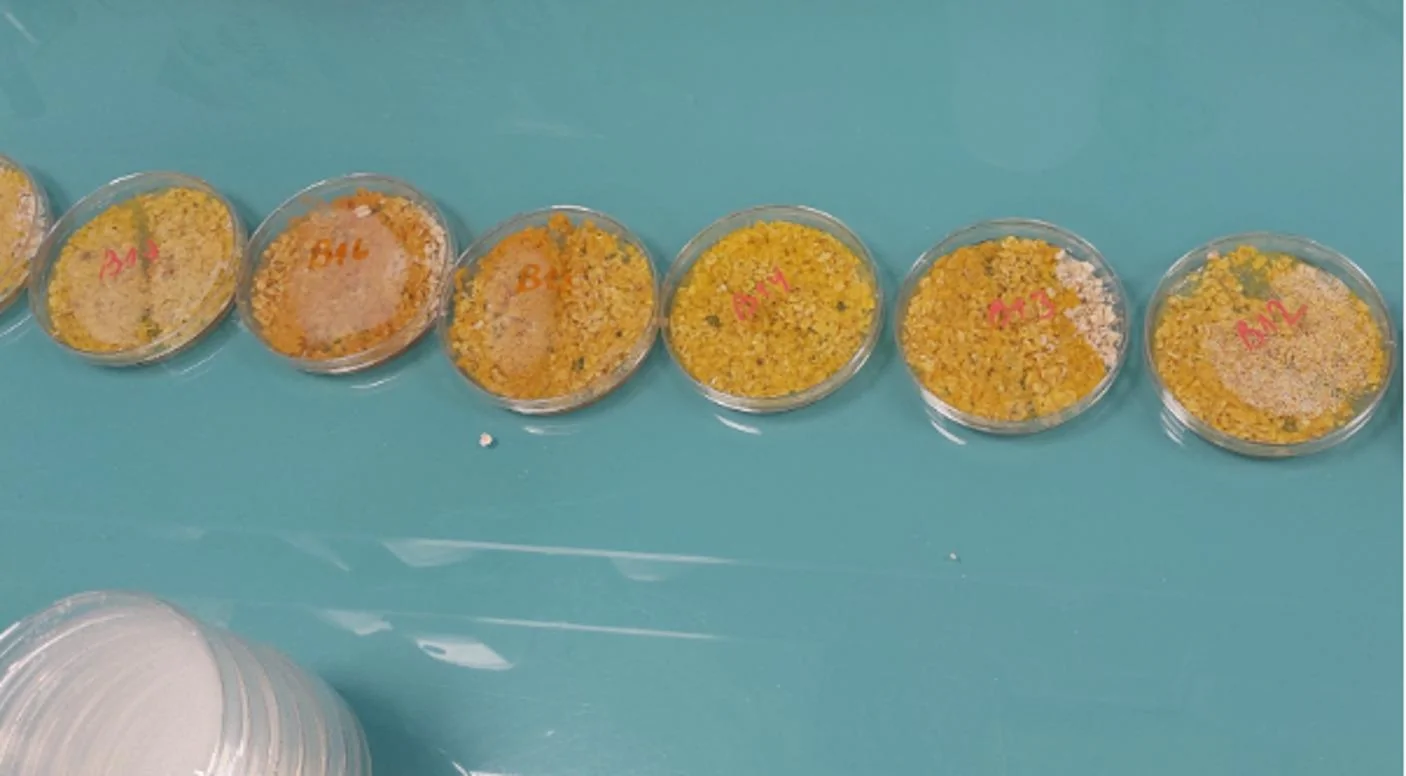
Petri dishes with *Physarum polycephalum* growing on oats, Center for Research on Animal Cognition, Toulouse

In the wild *Physarum* looks like yellow goo spilled over the forest floor. It crawls over rotting tree trunks, feeding on bacteria, fungi spores, and dead matter. As it forages it creates networks of protoplasmic tubes that connect its food sources and leaves behind a trail of slime that functions as a form of external memory, a map of places it has already visited (Reid et al., 2012; Sims & Kiverstein, 2022). It moves too slow for humans to notice; its pulsating movement becomes perceptible to us only on time-lapse videos and for this reason its behavior is studied in laboratory settings, where it is easy to cultivate and manipulate, rather than in the field. Depending on the research project and research questions *Physarum* may be grown on pure agar and fed porridge oats or on an agar medium already containing necessary nutrients. It does not require sterile environment nor does it pose any health risks, which makes it easy to cultivate outside of biological laboratories (Fig. 1).

Those peculiar characteristics have turned *Physarum* and several other slime molds into model organisms in the fields of cellular biology and biophysics, especially in the studies of cell aggregation and emergence of multi-cellular lifeforms (Aoki et al., 2022; Fleig et al., 2022), as well as in research on basic forms of intelligence and cognition (Awad et al., 2023; Boisseau et al., 2016; Dussutour, 2021; Dussutour et al., 2010; Mayne et al., 2015; Vallverdú et al., 2018), and decision-making mechanisms in unicelular organisms (Beekman & Latty, 2015; Latty & Beekman, 2010; Meyer et al., 2017). Matthew Sims refers to *Physarum* as an outlier or atypical model organism as opposed to more typical ones, such as fruit flies or *E.coli*, and demonstrates that due to its peculiarities *Physarum* poses new questions for philosophy of biology, particularly regarding the notions of individuality, niche consctruction, and cognition (Sims, 2024).

Due to its emergent properties and ability to form efficient networks the slime mold came to serve as a model and a metaphor of self-organisation becoming one of the most researched organisms in the field of biocomputing, as explained by Jeff Jones in his book-length study of *Physarum*’s computing abilities:

Many of the attractive features of non-classical computing devices (redundancy, fault tolerance) are thought to be based on the mechanisms of self-organisation and emergence, and the study of these properties is useful not only from a computational perspective, but also from a biological viewpoint—since much of the complexity in living systems appears to be built upon these principles. (Jones, 2015, p. 20)

*Physarum*’s computing abilities have turned it into a source of abstract network models, explored in a wide range of studies on network optimisation (Dirnberger & Mehlhorn, 2017; Fessel et al., 2012; Fricker et al., 2017; Jones, 2010; Sun, 2018). It has also been used as an artistic medium in bio-art projects that approach it as both a living organism and an abstract machine to explore the processes of emergence of human and non-human networks (Barnett, 2019; Schubert et al., 2015).

### Boundary object

In this paper I trace how network models and the concepts associated with them travel across disciplines and how, by defying our intuitive understanding of what an organism is and can do, *Physarum* serves as a boundary object that fosters translations between them. The concept of boundary object has been developed by Susan Leigh Star who offers the following definition:

objects that inhabit several social worlds and satisfy the informational requirements of each of them. Boundary objects are objects which are both plastic enough to adapt to local needs and the constraints of the several parties employing them, yet robust enough to maintain a common identity across sites. They are weakly structured in common use and become strongly structured in individual use. These objects may be abstract or concrete. They have different meanings in different social worlds but their structure is common enough to more than one world to make them recognizable, a means of translation. The creation and management of boundary objects is a key process in developing and maintaining coherence across intersecting social worlds. (Star & Griesemer, 1989, p. 393)

Those qualities of boundary objects turn them into tools for translation of concepts and practices between different disciplines and “social worlds”. One of the premises of the argument presented in this paper is that due to its peculiar characteristics as an organism and an object, *Physarum* is particularly keen to travel across disciplines, since its ability to create emergent structures that are abstracted into networks and optimising algorithms has turned it into a device that mediates between epistemic practices in different social fields. Leigh-Star understands this process as consisting in an assemblage of practices (of mediation, triangulation, standarisation) that aim at reconciliation of the different meanings that an object takes in different social worlds (Star, 2010, p. 609). Her focus is sociological, in that she is interested in the social ecology of objects, actors and practices in which this process occurs. In the following sections I focus instead on the content of those translations; my aim is to analyze the strategies of transfer of *Physarum* networks between biology, computer science and art, and the methods of arriving at (or eschewing) equivalence between *Physarum* and human networks. I employ Star’s concept to demonstrate how *Physarum* networks mediate between different disciplines and their epistemic practices and to identify the conceptual operations that make translations between them possible. I focus on network modelling practices in which *Physarum* is used as a source of network graphs and algorithms in order to examine the interplay of different senses in which it is a model (a model organism, a scientific model, an artistic medium) and how they are conceptually connected by means of different modes of comparison between *Physarum* and human networks.

### Outline of the argument

I approach *Physarum polycephalum* as a peculiar organism that straddles the line between single-celled and multicellular life, and examine its properties as an atypical model that does not fit the standard definition of model organism yet nonetheless serves as a source of generalizable insights into a variety of processes in biology and beyond. In the following sections I discuss *Physarum* as a biological model and examine how—due to its capacity to be abstracted into network graphs—it is adapted by other disciplines, namely, network science and art. I define abstraction as a practice of material translation, and argue that this practice allows *Physarum* to travel between biology, network science, and art, a process which is analyzed using Star’s concept of the boundary object. The analysis is based on three cases of interdisciplinary projects that use *Physarum* to model human infrastructures; the paper’s aim is to trace how the work of abstraction produces comparisons between slime mold and human networks via three distinct conceptual operations: analogy, metaphor, and equivocation. I conclude that these three modes of comparison are not mutually exclusive but complementary strategies that *Physarum* mobilizes as it moves between disciplines and I further argue that the projects under discussion counteract what Anna Munster calls “network anesthesia,” pointing towards a relational “aesthesia of networks” (Munster, 2013), that is, rendering them as a lived, more-than-human experience.

## Atypical model

### Model organism

According to Sabina Leonelli and Rachel Ankeny the key features of model organisms that distinguish them from other experimental organisms include their wide representational scope that allows for generalisations of research results far beyond one species: they “serve as the basis for articulating processes that are thought to be common across all (or most) other types of organisms, particularly those processes whose molecular bases can be articulated” (Ankeny & Leonelli, 2021, p. 7), which means that they need to be genetically standardized and provide easy access to the molecular mechanisms underlying the phenomena under study. They are also characterized by their specific representational target:

Model organisms explicitly represent whole organisms; they simultaneously allow access to specific processes and are investigated using a range of disciplinary approaches with the intention of integrating these approaches to develop a multi-level understanding of their evolution, structures, and behaviours. In contrast, experimental organisms are models for specific phenomena, to be investigated through a particular discipline or approach with its accompanying set of techniques and practices. (Ankeny & Leonelli, 2021, p. 9)

*Physarum polycephalum* is usually not included in the short list of model organisms,^2^ and it does not fully match the definition provided by Ankeny and Leonelli. Nonetheless, Sims calls it an atypical model organism and provides an analysis of *Physarum*’s role in biological research that extends far beyond what Ankeny and Leonelli ascribe to experimental organisms. The focus of this section is on the particular characteristsics of *Physarum* that allow to characterize it as both a model organism and a network modelling device.

*Physarum* can be obtained in a standardized form from biological supply companies but the standardisation results from its peculiar biology and not from genetic intervention: its body can be cut into pieces and form separate bodies of identical genetic makeup. It is genetically stable, since a cell with different mutations in different nuclei would be less likely to survive; however, for the same reason, namely, due to the large number of nuclei in the cell and the fast growth of the DNA mass, its genetic mechanisms remain hard to study and even more difficult to manipulate.^3^

**Fig. 2:**
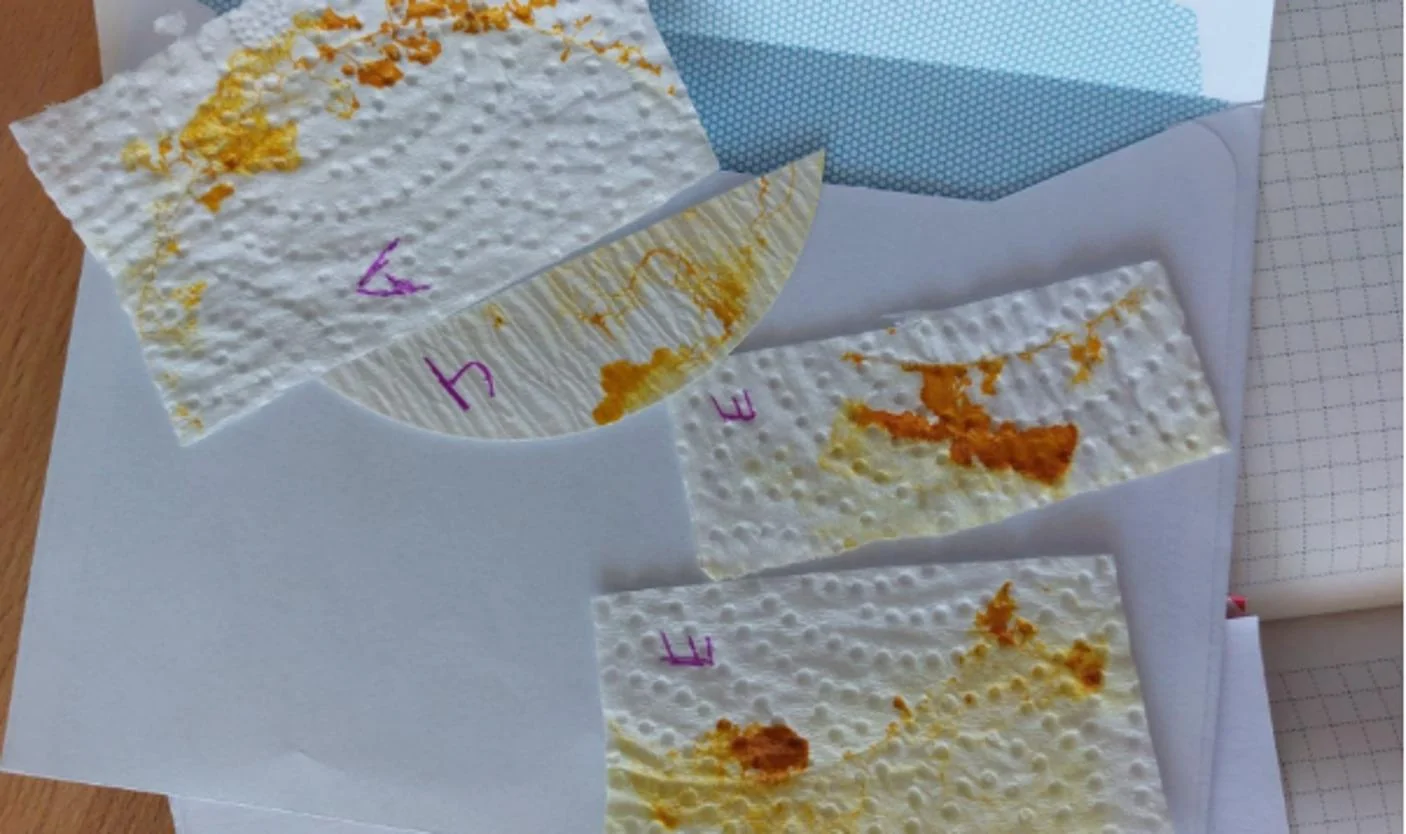
*Physarum* sclerotia from three different sources shared with the author by artist Heather Barnett

Another characteristic of model organisms is their situatedness within particular forms of social organisation of research practices in which they have been developed, and which Ankeny and Leonelli have named “model organism repertoires” (Ankeny & Leonelli, 2021, p. 38). Since those repertoires depend on elaborate and expensive research infrastructures they are relatively stable and have clear boundaries; model organisms are genetically developed and standardized to serve a particular purpose within such a repertoire. In contrast, *Physarum* plasmodium is routinely shared in an informal way among researchers, artists and lay persons, spreading far beyond biological laboratories.^4^ The strains that circulate among those groups are often very old, since plasmodium can be dried out and later revived when needed. Often research is done on one specimen of one strain, because plasmodium can be divided into smaller parts almost infinitely. It can be claimed that instead of being transformed into a model by means of genetic modification, *Physarum* has evolved into a laboratory organism as a result of practices of experimentation and sharing among researchers (Fig. 2).

One of the crucial characteristics of *Physarum* is that it exists at the threshold between single celled and multicellular life-forms. Peter Godfrey-Smith’s distinction between an organism and an aggregate could be used here to argue that in certain important contexts *Physarum* is both, and that this double characterisation makes it so versatile and easy to transfer between disciplines. Godfrey-Smith points out that this distinction is useful to think about human forms of social organisation:

… we can distinguish more organized systems from more aggregative ones. More organized systems include cells and organisms, and more aggregative ones include populations of those organisms. There are intermediate cases, like honey bee colonies. (…) Mechanistic analysis is most appropriate when dealing with organized systems. Aggregative ones are better described in terms of tendencies that arise from the combined action of parts which each have some degree of independence. The two kinds of systems occur at different scales; there are size constraints, it seems, on highly organized systems, due to the difficulty of keeping the parts working together, and organized systems often have distinctive kinds of histories. (…) It is interesting to think about human societies, which can be very large objects, in terms of this distinction. (Godfrey-Smith, 2013, p. 18)

*Physarum* plasmodium is an aggregate of individual cells that form a structure that is highly malleable and does not have a fixed form or state—which distinguishes it from other multicelular organisms. In my view, here lies its peculiarity as a model organism: on the one hand, it shares some crucial characteristics with typical model organisms, such as easy cultivation, standardisation, as well as the possibility to extrapolate research results onto larger classes of organisms, which make it a model in studies on cell motility and aggregation, biological network formation and basal cognition. On the other, due to its self-organising and emergent qualities it lends itself to various practices of abstraction that produce network models, which means that the results are not generalisable onto other organisms but onto their aggregates (human groups) as well as non-biological phenomena such as telecommunication networks. This dual character of an organism and an aggregate is what turns it into an effective boundary object mediating between disciplines, since it can be used to explore both organized and aggregative systems.

The debate on the epistemic functions of model organisms has focused on their representational and non-representational aspects, which partially reflects wider debates on the epistemic functions of models in science (Godfrey-Smith, 2006; Knuuttila, 2011; Knuuttila & Loettgers, 2021). The representational approach assumes a necessary correspondence (understood as similarity, structural analogy etc.) between the model and the target system, which makes representation possible. Non-representational approaches define models as artefacts that serve as epistemic tools whose construction and manipulation provides new theoretical insights (Knuuttila, 2011, p. 263); Morgan and Morrison treat them as “autonomous agents in the production of scientific knowledge” (Morgan & Morrison, 1999, p. 39). In the case of model organisms their epistemic function has been discussed by a number of scholars who approached them as scientific representations (Sartori, 2023), surrogates for target systems or exemplars of wider classes of organisms (Bolker, 2009), analogue simulators (Tee, 2019), and as laboratory artefacts and epistemic tools (Ankeny & Leonelli, 2021).

It seems safe to assume that model organisms have both representational and non-representational aspects and the point is to define the relation between them. Ankeny and Leonelli argue that the non-representational are conditioned upon the representational, since model organisms can be useful as research tools only if they are assumed to represent the phenomena they model, which will also be the case of *Physarum* network experiments. Sim-Hui Tee makes an argument for defining them as analog simulators based on the observation that model organisms do not adequately represent their targets because of their dynamic properties, namely, the changes induced by heterogeneous factors whose mechanisms cannot be reproduced in target systems, as well as the fact that many of those changes have emergent characteristics, which means that they are not deducible from the state of the sum of the elements of the system. Conceptualising their epistemic function as analog simulators allows to go beyond the notion of model organisms as static representational systems and take into account their dynamic and emergent properties, which in the case of *Physarum* is one of its crucial and most appealing characteristics.

It could also be argued, that the very distinction between representational and non-representational functions stems from the assumed divide between theory and reality, which in the case of biology is difficult to uphold, as demonstrated by Evelyn Fox Keller in her analysis of computational metaphors in genetics. She points out that defining models as “tools” is itself a representation (a metaphor) and instead of distinguishing between representation and instrument, she argues that representations also do conceptual work, since there can be different representations of the same thing that serve different purposes. By examining the metaphor of “biological computer” she demonstrates that representations are a particular type of tools able to transform the objects they are used to study and that metaphors guiding the research process can take material form (Keller, 2000, p. 84).

Network models extracted from *Physarum*’s protoplasmic tubes have both representational and instrumental functions, while the relation between them is shaped by the underlying assumptions of a given modelling project. In the case of the projects that use *Physarum* to model human networks the correspondence that is the basis of representations is not given but achieved by means of abstraction. Representations so understood are not only tools, but also artifacts produced in the process of translation between the slimy and the human. Therefore, before I proceed to investigate this relation it is necessary to step back and consider the process of abstraction of networks from the slimy tangle of protoplasmic tubes.

### Abstraction as material translation

When models are understood as representations of their target systems they are based on a structural analogy between the domains that is assumed from the outset, while abstraction as part of model constructon means simplification, omission of irrelevant features, whose ir/relevance is established in advance. However, when a model is understood as a system in and of itself it is possible to think of abstraction as an exchange of features, an open-ended process of material translation guided by certain concepts, narratives, and metaphors. Such notion of abstraction has been proposed by a number of scholars (e.g. Carrillo & Martínez, 2023) and most recently by Tarja Knuuttila, Hanna Johansson, and Natalia Carrillo, who argue that abstraction is embedded in particular artistic and scientific practices and involves material translations, that is, substitutions of features: “Through the use of different representational and experimental artefacts, material translations enable scientists to arrive at abstractions. As such abstractions usually do not display the underlying material and semiotic work” that goes into their creation (Knuuttila et al., 2024, p. 192).

Following David Landy, Knuuttila et al. further argue that contrary to the common view of abstraction as omission of irrelevant features aimed at isolating and revealing the underlying structural relations, abstraction is a material process more accurately characterized as managing of features to establish relations, or, in Landy’s words, “to get relational work done” (Knuuttila et al., 2024, p. 204). And since it may involve multiple substitutions it can lead in multiple directions, depending on which features are deemed relevant. Carrillo and Martínez examine the role of metaphors in establishing the criteria of relevance in scientific modelling and suggest that abstraction occurs along certain paths organized by metaphors:

the path from metaphor to model is closely related to the way in which a metaphor can guide the development of concepts through a process of interpretation of theories and (experimental) practices, within the context of a research program. This guidance, in turn, is closely related to the way the cultivated metaphor is able to help us organize different experiences as part of a credible narrative. (Carrillo & Martínez, 2023, p. 253)

It follows that metaphors are not just representations but crucial instruments in the material processes of abstraction and model creation. This notion of abstraction as guided by metaphors will underlie the analysis of the *Physarum* network projects discussed in the following sections. The models I will focus from now on will be the abstract networks extracted from *Physarum*’s morphology and their underlying nrarratives, however, it should be emphasized that *Physarum*’s material affordances—its qualities as an organism and a biological model—provide the ground for the production of these narratives.

The notion of abstraction as material translation allows to characterize *Physarum* as a modelling device and an analog simulator that can be used to produce different abstract models depending on the metaphor or narrative guiding a particular project. This openess to translation is also the feature that turns it into an effective boundary object. In the work of Leigh Star translation remains vaguely conceptualized and could be understood as a metaphor in the sense that it does not denote a literal translation but a form of mediation between different social codes, a search for equivalents and approximations. The concept of material translation—a process of substitution of features that is guided by particular representations—can be applied to the work performed by both *Physarum* and the networks abstracted from it as boundary objects, translating between disciplines. In the following sections I will argue that abstraction of networks from the *Physarum*’s body is the means of translation between the slime mold and the human, and fosters mediation between disciplines. Their highly abstract character opens them up to a variety of concepts and narratives in which the comparison between slime mold and humans can be grounded, as well as to different methods of comparison, such as analogy, metaphor and equivocation.

### Analogy, metaphor and equivocation

Analogical reasoning in science is based on the assumption of comparability of structural elements between two domains. As explained by Knuuttila and Loettgers “analogy enables the mathematisation of the target domain in terms of relational generalisations that may yield abstract schemas common to both source and target” (Knuuttila & Loettgers, 2014, p. 78); it allows to compare the structure of relations within both domains. Such is the case of *Physarum* network experiments, which rely on structural analogies between the abstract networks to which both human and slime mold transportation/communication/distribution systems can be reduced. Analogy is based on an assumed similarity or equivalence, which is inferred from the resemblance of human and *Physarum*’s networks, once they are recreated on an agar plate. If the relation between slime mold and human networks is defined as analogy, there should be a clear relation between source and target domains, since in scientific models based on analogies the source domain is the more familiar one and the target domain—the unfamiliar one being explored by means of the familiar. However, in the case of *Physarum* network projects this relation is uncertain—it can be argued that the slime mold is the unfamiliar element used to explore the familiar and defamiliarize it; the similarity between them is not assumed but produced in the research process.

While analogies allow for an exploration of an unfamiliar domain by comparing it with a familiar one and identifying structural elements or patterns common to both, metaphors often serve to explain the abstract in terms of the concrete: as argued by Lakoff and Johnson in their seminal work, this is why we can think of love as a journey but not necessarily of journey as love (Lakoff & Johnson, 1980, p. 115). Moreover, in their theory, metaphors are always underwritten by a particular ontology; there are no standalone metaphors, nor ones which are not based on shared ontological premises. As indicated above, metaphors not only illuminate similarities but establish them by creating criteria of relevance for the work of abstraction, deciding which similarities count and bringing their referents together. Catherine Elgin claims that metaphor can go further than that and become “…a device for identifying previously unnoticed classes, and for stating previously inarticulable truths. It equips us to see new likenesses and differences, patterns and discrepancies both within and across domains” (Elgin, 2002, p. 18). While analogy captures formal or structural similarities between domains, metaphor allows for an asymmetry in that the domains do not need to overlap to be compared.

One of the most radical accounts of the asymmetry of metaphor can be found in Graham Harman’s discussion of Ortega y Gasset’s essay on the subject (Harman, 2018). Harman’s argument regards the irreducibility of things to their properties; in his account, metaphors work at the limits of comparison and point to the incomparable. In Harman’s reading of Ortega, resemblance between things is superficial and misleading as to the real working of metaphor, which “satisfies us precisely because in it we find a coincidence between two things that is more profound and decisive than any mere resemblance” (Gasset & Silver, 1975, p. 19). Metaphor then, is not “an assimilation of real qualities between two things” (Harman, 2018, p. 73), but a device that gives us indirect but real access to “things in themselves”. In metaphors we can perceive the gap between the thing and the sum of its properties, that is, the space of incommensurability and incomparability.

Thus, metaphors may serve as means of exploring neither differences nor similarities, but that which is left out after all similarities and differences are exhausted. The reason Ortega’s chosen example—of a cypress being a flame—does work as a metaphor is because “the similarity of linear outline in cypress and flame is so extrinsic, so insignificant with respect to each component, that we do not hesitate to call it a pretext” (Harman, 2018, p. 20). It would follow, that contrary to salient similarities between domains that form the basis of analogies in scientific reasoning, metaphors can be based on just a pretext of similarity because their aim is not comparative but creative. According to Harman, Ortega “establishes the basic mechanism of metaphor as an inessential likeness that serves to fuse two vastly dissimilar entities into an impossible new one” (Harman, 2018, p. 74). If we consider the function of metaphor so understood as defining a path of abstraction, obvious similarities would constitute a criterion of irrelevance and the process of abstraction would aim at establishing new relations between dissimilar objects—which is a common function of abstraction in art more often than in science.

Harman’s concept of metaphor significantly differs from Lakoff’s and Johnson’s in that it does not assume any prior ontological structure that is expressed in metaphors, but rather defines metaphors as means of an infinite ontological creativity made possible by the irreducible ‘inwardness’ of things. In his essay, Ortega argues that any metaphor is based on the assumption, that things are “not correlates of each other, but [have] equally rich independent lives” (Harman, 2018, p. 67). In Ortega’s words, “everything, from a point of view within itself, is an I” (Gasset & Silver, 1975, p. 15), and even though this ‘I’ cannot be accessed by means of reasoning, it can be perceived through art: according to Ortega “The aesthetic object is inwardness as such—it is each thing as ‘I’” (Gasset & Silver, 1975, p. 16).

In classical philosophy of science the asymmetry of metaphor is epistemological—the known explains the unknown. According to Lakoff, the asymmetry is structural: the concrete explains the abstract, not vice versa. According to Harman, the asymmetry is ontological: “the assumption of a ‘vice versa’ at work in metaphor is a fatal error” because it obscures what he calls “the deep divide or tension between an object and its qualities” (Harman, 2018). This tension points to the potential of untranslatability or failed translation, which resonates with the concept of controlled equivocation theorized by anthropologist Eduardo Viveiros de Castro. In his account of Amerindian perspectivism to have an ‘I’ does not refer to any kind of inwardness, but to the ability to occupy a point of view. Ultimately, it may not matter whether the black box can be decoded or the obscurity of things-in-themselves shed light on, as long as what remains in the dark provides an exit from what de Castro calls the “stifling bedroom of the Same” (Castro & Skafish, 2014, p. 42), which consists in “always seeing the Same in the Other, […] thinking that under the mask of the other it is always just ‘us’ contemplating ourselves” (ibid.). Whereas Harman sees the way out of this solipsism in the things’ inaccessible inwardness, for de Castro it leads through “controlled equivocation,” which he defines as a “referential alterity between homonymous concepts” (Castro & Skafish, 2014, p. 87), in the case of *Physarum* the main equivocal concept being the network, which refers to both the material protoplasmic tubes and their abstract representations, but, as we shall see in the following sections, other concepts and metaphors that guide *Physarum* projects are also equivocal because of the incommensurability between the human and the slimy modes of existence.

## *Physarum* network models

### Optimisation

The uncanny abilities of *Physarum polycephalum* have been made famous by Toshiyuki Nakagaki who won his first IgNoble prize for putting the slime mold in a maze from which it managed to find the shortest route of escape (Nakagaki, 2001, 2005). Nakagaki and his team proceeded to examine the slime mold networks as well as its decision making process and thus opened a new research avenue that was followed by many others (Aoki et al., 2022; Fricker et al., 2017; Nakagaki & Guy, 2008; Tero et al., 2006, 2010). *Physarum*’s protoplasmic tubes that are able to shift and adapt to changes in the environment have inspired a large body of research that aims to formalize them into networks, analyze their properties and apply them as models in other areas (Adamatzky & Jones, 2010; Jones, 2010; Tero et al., 2006). The most famous *Physarum* network experiment was performed by Nakagaki and his team in 2010 (Tero et al., 2010; Watanabe et al., 2011), winning him his second IgNoble. *Physarum* was put on an agar plate which contained a map of the towns around Tokyo represented by oats; it then fairly precisely recreated the railway network that connects them. Based on the analysis of the actually existing railway network and its slimy approximation an adaptive network model with adjustable parameters was proposed, which emulated the experiment’s end product.

All those studies are based on the material practice of abstraction of network patterns from *Physarum*’s body, which I will describe using the example of a project I had the chance to observe in the Center for Research on Animal Cognition in Toulouse in April 2024. The project’s aim was to build a predictive algorithm that could extract *Physarum*’s networks’ qualities into a model for optimising actually existing internet infrastructure and was pursued by biologists from the Center in cooperation with the programmers from an internet start-up company (Gourjade-Gosselin, 2024). The analysis was focused on the comparison between two species of slime mold, *Physarum polycephalum* and *Badamia*, and the particular parameters of their networks, such as cost effectiveness, robustness and efficiency.

**Fig. 3:**
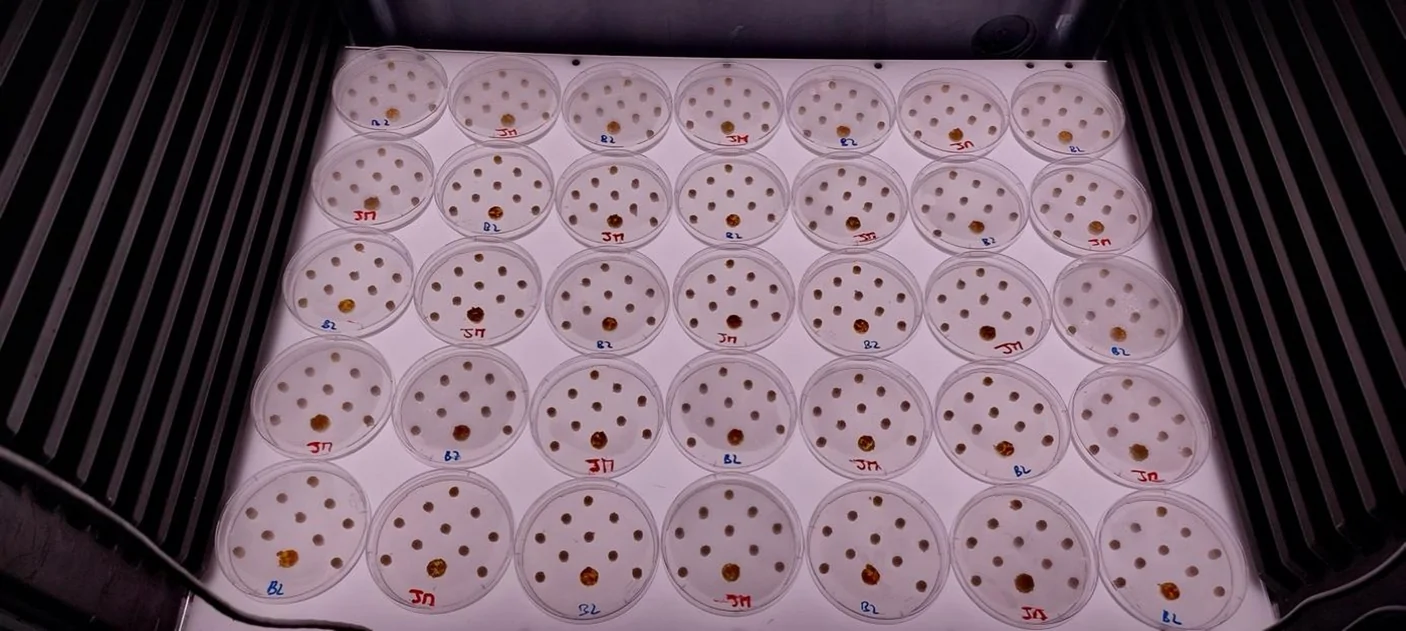
Petri dishes with a distribution of network nodes and two species of slime mold, Center for Research on Animal Cognition, Toulouse

The networks created and analyzed within this project were distributed over a pre-given constellation of nodes and captured 48 h after inoculation; the project did not include an analysis of their temporal aspect. 700 dishes were prepared with different configurations of nodes and different placements of the slime mold (5 different versions of the experiment), and 700 images of slime networks extracted and analyzed. In one experiment the node placed the farthest from the slime mold consisted of oats combined with egg yolk, which is a more attractive food source for the slime mold than pure oats. In another, the central node was removed after 48 h and the reconfiguration of the network captured after further 15 h. The work of abstraction started with the tedious and time consuming labour of preparing the agar Petri dishes and the oat-based nutrient (standardized into equal units to avoid bias), distributing the nutrient on the dishes according to a pregiven pattern, inoculating each dish with a slime mold, putting them in an incubator in a particular order, setting up time-lapse cameras, extracting the results into a computer program that can analyze them, straightening *Physarum* lines into network graphs, manually checking the correctness of the extracted networks one image at a time, using an algorithm to identify and analyze extracted network patterns and, finally, describing them by a limited number of parameters. In this process any anomalous behavior such as *Physarum* leaving the petri dish and wandering around the incubator walls were left out, since the criteria of relevance were agreed upon in advance and determined both by previous research practices and the objectives of the project (Fig. 3).

The aim of the project was to create an abstract model based on the registered parameters of slime mold networks, which would then be translated into possible optimising strategies for human telecommunication infrastructure. In this case the process of abstraction is guided by an analogy between slime mold and telecommunication networks and the slime mold serves as an analog simulator. *Physarum* networks are described in economic terms: the parameters measured in the experiment were efficiency, cost, robustness and the trade-offs between them. The parameters of slime mold networks are assumed to be applicable to human-made telecomunication networks because of the underlying assumption of structural and functional resemblance between both, which in turn is based on the previous assumption that the protoplasmic tubes have similar structures and functions as internet networks.

It can be argued that the analogy between human and slime mold communication systems is produced in the process of abstraction of network models from the slime mold’s protoplasmic tubes. Similarly to other network models, they “operate at a high level of abstraction, which makes them independent of the (often very complex and unknown) mechanistic details of the systems they are supposed to represent” (DiFrisco et al., 2025, p. 158). This allows to describe their general properties such as robustness, but leaves out both the causal and temporal factors, that cannot be accounted for without bringing those mechanistic details back in.^5^ This obfuscation of causal mechanisms that underlie network creation, the “mismatch between abstract and idealized network-level models and the dynamic causal mechanisms they represent” (DiFrisco et al., 2025, p. 160), makes for their wide applicability and low explanatory power, since the same network structure can be a result of many different processes.

According to Knuutila and Loettgers “both structural network models and dynamical systems can be thought of as defining and generating abstract spaces of possibilities, parameterized by the modeled system’s features, that is, the specific formalisms, variables, and parameters that those models are built upon” (DiFrisco et al., 2025, p. 160). In the case of *Physarum* projects this abstract space starts with the empty agar Petri dish, a space devoid of any environmental features that determine both *Physarum* and human networks. This image is then projected onto human made-networks: it feeds the assumption that human-made networks operate in empty abstract spaces like the slime mold on a Petri dish into the model. Thus the analogy between both types of networks is achieved in the process of material translation that is guided by the economic metaphors of network efficiency on the one hand, and the abstraction of social space in which internet networks operate on the other.

### Colonisation

In other studies that use *Physarum* networks to explore human transportation networks, abstraction is guided by more elaborate narrative frameworks, as in the case of the works of Andrew Adamatzky, head of the Unconventional Computing Lab in Bristol, one of the most prolific researchers using *Physarum* to tell human stories. Having initiated a variety of projects that explore the creative potential of the slime mold at the intersections of computer science and art, Adamatzky actively connects artists and scientists, fostering interdisciplinary collaborations and hosting artistic residencies in his laboratory (Adamatzky, 2019; Adamatzky et al., 2013). His penchant for speculation and working with unconventional computing substrates (not only slime molds but also fungi and colloids) has led him to engage in research that blurs the line between science and science fiction (e.g. the Physarum Chip project,^6^ a consortium founded to invent a computer chip operated by the slime mold).

Adamatzky has performed a large number of experiments recreating existing human transportation systems and simulating possible future ones with the help of *Physarum*. Those experiments include a temporal component in that they trace the process of expansion of *Physarum* on agar plates simulating various scenarios: from global road formation starting in Beijing, to hypothetical colonisation of the Moon (Adamatzky, 2012, 2016; Evangelidis et al., 2017), to the simulation of human migration routes across the US-Mexico border (Adamatzky & Martinez, 2013). Thus the slime mold’s meandering around the Petri dish gains a narrative dimension and is used to tell stories of human collective movement which results in road formation, a process framed as either colonisation or migration. Colonisation in this case refers to *Physarum* spreading over Petri dishes, even though it is not the term used in biology to describe this process. By “colonising” the dishes the slime mold simulates possible scenarios of road formation, blackboxing the social, economic and political processes that underlie the formation of human transportation systems and conflating colonisation and migration into one natural phenomenon. In this context migration and colonisation work as metaphors because the projects are underwritten by a a set of ontological assumptions, which serve as the ground for comparison, rather than structural analogies between human and slime mold networks.

The agar plate serves as a map with oat distribution corresponding to major urban centres, the slime mold’s movement on this surface is then projected back onto human space^7^ and forms the basis for inferences about collective human behaviors. However, the recreation of human networks does not start with the slime mold connecting the nodes but with creation of an empty space in which it operates: the experiments simulate colonisation of this space, that could be defined as a *terra nullius*, emptied out and ready for invasion. The smooth empty space allows to project man-made systems onto it and thereby produce the convergences or analogies between slime mold and human transportation networks: it is the ground on which analogies and metaphors exploring connections and similarities between humans and the slime mold can be built.

The colonisation metaphor draws attention to the fact that *Physarum*’s protoplasmic tubes stand in for the networks of extraction and transportation of resources and the calculation of the shortest path between the nodes is not a just mathematical exercise but also an exercise in resource management. In contrast to the optimisation project described above, Adamatzky’s experiments do not use economic terms to describe *Physarum* networks, but instead embed them in a particular political ontology, which assumes the existence of empty territories, conflates migration with colonisation, and positively valorizes the latter. In effect, the experiments render both colonisation and migration as a natural and emergent process rather than socio-political phenomena.

At the same time the metaphors of migration and colonisation point to the incommensurability of the processes of human and slime mold movement rather than structural similarities between them. Engaging *Physarum* as a model for socio-political processes produces strange effects: the image of a yellow blob crawling over Petri dishes and connecting pre-given nodes oscillates between an idealized harmonious collective movement and an alien blob swallowing up everything in its wake. Instead of modelling human social phenomena to make them more familiar, it defamiliarizes them by rendering them in slime. *Physarum* becomes a narrative medium, an instrument for telling human stories and its double nature of organism and aggregate makes for the ambivalence of the stories and images it produces.

### Self-organisation

Adamatzky’s experiments are situated at the intersection of computer science and art and have inspired a number of bio-art projects that explore *Physarum* networks to tell other stories about human social organisation, in which the slime mold’s activity is framed not as an act of colonisation but as a research tool to explore horizontal forms of social organisation and collective resource management. In the narratives that accompany those projects the relation between humans and slime mold constitutes an explicit object of inquiry and the networks act as mediators between them. Examples include Heather Barnett’s “Being slime mold” where *Physarum*’s behavior is abstracted into an algorithm which then allows to investigate patterns of collective human movement and exploration of space, as well as Jonathon Keat’s project “Plasmodium Consortium” (2017–2018) which was based on the premise that through the intensification of global connections humans have effectively become a super-organism and need *Physarum* to teach us how to act like one. Keats defines the slime mold as a super-organism (which in biological terms it is not) and argues that due to its ability to behave intelligently as an aggregate it could serve as an impartial arbiter in human affairs (Plasmodium Consortium, 2018)—super-organism serves here as a metaphor that allows to produce equivalence and thus a possibility of translation between slime mold’s and collective human behavior.

Here I discuss another example of art projects modelling human self-organisation with *Physarum*^8^: the works of the media artist and computer programmer Sarah Grant, whose principal medium is network technology. For Grant computer networks are complex and layered technologies that operate as a black box; she sees her work as demystifying them and exposing the mechanisms of their operation. She’s particularly interested in exploring the materiality of networks which often gets obfuscated by wireless technologies:

I like thinking about computer networks in terms of other materials, and in terms of organic material as well. Because computer networks are typically thought of as being those intangible wireless things, sort of in the air, it is definitely not organic (…) to think of networks in terms of the slime mold, which is a live creature and its bright yellow, it’s fragile but also resilient in other ways—it is such a different way to think about networks.^9^

Her collaboration with *Physarum* was inspired by the Tokyo railway experiment: “It was amazing to see a slime mold solve in a day what human engineers presumably took years to figure out.”^10^ In several projects investigating the topologies of *Physarum* networks she explored the modes and methods of comparison between them and human networks (Grant & Savić, 2020; Savić & Grant, 2022). In the installation *Physarum Topologies* exhibited in Art Laboratory Berlin in 2020^11^ she captured the movements of the slime mold over surfaces covered with diagrams of 8 different network topologies with oats distributed in places of nodes. The oats were dyed with food dye which allowed to follow the transportation of nutrients within each network. In Grant’s project the distribution of nutrients within the plasmodium is a metaphor of distribution of information, as the particles of the food dye can stand for information packages transmitted across internet networks. By investigating how the slime mold navigates the network topologies she hoped to gain insights into the mechanisms of distribution of information and resources across human networks. The installation was on show for several weeks and viewers could follow *Physarum* creating its networks in real time.

Grant is interested in exploring the actual and conceptual relations between slime mold and telecommunication networks:

… the relationship between computation and organisation is many-directional. Model organism—slime mould is doing the work of a computer model, but we do not expect to see predictions or realistic simulations. Slime mould computes, nevertheless, and communicates. The interest in the way it communicates supports our ability to move between the real world and its abstraction in a productive manner in our articulations of these experiments. (Grant & Savić, 2020)

Her aim is to explore not just the relation between the material body of the slime mold and its abstraction into networks but also to investigate the possibilities of comparing them without striving for establishing any strict equivalence. In another project on which she collaborated with the artist Selena Savić, the slime mold was used to model a horizontal resource distribution system and simultaneously explore the possibility of comparison between slime and human networks:

How would this be compared to communication in a housing cooperative? By carefully observing the flow of colour through the tubes with a microscope, we could trace the interaction of the slime mould body with food sources and areas of the dish. If we would decide to treat these flowing particles as messages or packets, we could find most optimal paths for transmissions in the different setups (centralized, sparse or regularly distributed nodes). But the point of the curious (even naive) attitude is to remain undecided about the exact translations (particles to packets) and articulate transformative ways to compare the slime mould processes to networked communication. (Grant & Savić, 2020)

Instead, by modelling community resource management with the slime mold Grant and Savić establish equivalence between computation, communication (or the flow of information) and human collective behavior and investigate the methods of translation and comparison between them. For them the value of *Physarum* network experiments lies in the space of indeterminacy they open, which is also the space of conceptual creativity in thinking about relations between slime mold and humans, as well as between computation and its substrates. In contrast to network optimising projects, art projects involving *Physarum* thrive on equivocation that is produced by refusal to precisely determine the terms of translation between the slime mold and the human.

In mathematics and network science the computational abilities of the slime mold are exploited by means of abstracting the computation process from its substrate and including it in other processes by extracting graphs and algorithms. When *Physarum* is used by artists, computation is not the means to an end, but an end in itself and the object of study. As observes the artist Miguel Carvalhais, computation, regardless of its substrate, “is behaviour, action and being, and cannot exist as something static. Computation is becoming, something permanently turning into something else, perpetually transforming and metamorphosing” (Carvalhais, 2022, p. 23). As such, computation is about the future, so even though, as mentioned by Grant, *Physarum* does not provide predictions or realistic simulations, computing with the slime mold fosters imagination of possible human futures, which in turn necessitates embedding the computation in a narrative framework. *Physarum* models, whether in network science or bio-art, can foster narratives that allow to see human societies as collective organisms in the state of perpetual becoming with inherent optimisation mechanisms for information and resource distribution.

Grant’s initial premise is the observation that networks manipulate us and as such they are agents—whence the intuition that the living networks of the slime mold can shed some light on how other networks operate, as their common denominator and the ground for comparison is their aliveness and agency. According to Carvalhais, “Algorithms and code are deterministic and look back, but once they are deployed as computations, they start manifesting emergent and generative phenomena because computation looks forward, opens new fields of action and creates unprecedented forms and behaviours” (Carvalhais, 2022, p. 24). The art projects engaging *Physarum* networks capitalize on computation’s potential of generating new possibilities and states of affairs.

Grant’s experiments emphasize the agency of networks and by projecting the slime mold onto internet networks render the latter alive. In her work *Physarum* points to the experience of networks as producing and organizing human relations, but also as an aesthetic experience in the sense of the capacity of being affected. Networks consist not just of nodes and lines, but of the underlying work of abstraction, that is the work of producing relations that the networks are supposed to represent. Anna Munster argues, that the ubiquity of network models has numbed us to how they affect us (Munster, 2013). Artists such as Grant attempt to make us more attentive to the experience of networks, that is predominantly aesthetic—in Ortega’s sense of being confronted with their ungraspable inwardness, an experience that Munster has named an aesthesia of networks.

## Aesthesia of networks

When the relation between the slime mold and the human domain is framed as analogy, the focus is on structural and functional similarities between networks; their abstract nature and low explanatory power allow for far-fetched comparisons and can be understood as a manifestation of what Munster calls “anaesthesia of networks”:

A network image can be rendered for just about every aspect of day-to-day life for which data exist, including financial information, organizational data, mapping systems of every variety, social networks, and technical ecologies of all kinds. But such images have become uniform, dominated by links and nodes, visualized as direct lines connecting dots. The very sameness of this rendering, operating across all and any network, creates the idea of the network as infinitely transposable, in spite of what might be specifically visualized. (…) networks mirror networks, in a pervasive mimesis barely concealing a visual and conceptual slide into what I call network anesthesia—a numbing of our perception that turns us away from their unevenness and from the varying qualities of their relationality. (Munster, 2013, p.2)

The assumed transposability of networks is the basis of analogy between the slime mold and the human domain. The relation between source and target is clear—the slime mold is the source of network patterns that are projected onto human-made networks; even though the node distribution is determined by humans, as long as it is random or abstract it does not project an explicitly human narrative onto the slime mold. However, the black box function of networks—the fact that network models obscure the mechanisms that led to their creation, or in Munster’s words, the qualities of their relationality—turns the very concept of the network into an equivocation the moment the network model is used as an instrument of comparison between actually existing entities or processes.

When *Physarum* is used to model human behavior, the domains fall into a feedback loop, where the human domain is the source of network patterns recreated by the slime mold which are then projected back onto the human. Here the effect (if not always the explicit aim) of the comparison is not to make the unfamiliar familiar or to find a common denominator for both domains, but to render the human unfamiliar by comparing it to the slime mold and thus explore new possibilities of collective human organisation. In Adamatzky’s and Grant’s experiments the slime mold could be interpreted as a metaphor of colonisation or horizontal resource distribution, the latter being abstract terms demanding explication in terms of the concrete. Slime mold as metaphor invokes an underlying political ontology, whether colonial or communal: it can represent either a menacing colonising force or a process of emergent self-organisation.

However, if we look at the slime mold’s protoplasmic tubes which recreate roads drawn on a map, it becomes clear that the similarity is superficial and serves as a pretext for exploring *Physarum*’s curious behaviors, or, in Harman’s words, the non-human ‘I’ that is the function of its inwardness that can never be exhausted by its perceptible qualities. When the slime mold is forced to rehearse the same scenario of expansion, contraction, extraction and transportation, recreating the same pattern over and over again, in the juxtaposition of *Physarum* lines with maps of human transportation and communication systems a new entity emerges that fuses collective human activity and slime mold’s movements, turning the socio-political processes of colonial expansion or communal resource distribution into an object in Harman’s sense, endowed with a separate ‘I’ and an irreducible inwardness. Seen through the prism of *Physarum* network models, human forms of social organisation are rendered as aesthetic objects, endowed with an ungraspable interiority and an agency of their own.

The ungraspable aspects of the slime mold could be approached from the functional (black box) or ontological (thing in itself) perspective with their respective methods of comparison being analogy and metaphor. However, Grant’s attitude of refraining from searching for an accurate translation between the domains, which leaves a space for creative experimentation and openness to the unexpected, could be considered a third method of comparison, that is, equivocation. Her project of demystifying networks with the help of the slime mold produces the effect of rendering networks alive and sentient—able to affect and be affected. This resonates with Munster’s concept of network aesthesia, the ability of networks to experience and to generate new sensibilities, or in Deleuzian terms, the becoming perceptible of networks. According to Munster, this experience foregrounds dynamic relationality, which gets lost in the ubiquitous node-line network image, but reemerges in art, which is the method of “differentially and relationally encountering the imperceptible” (Munster, 2013, p. 10) and thus generating new network sensibilities, or a “radical aesthesis” of networks. Both Grant’s and Adamatzky’s projects engage the slime mold to generate new sensibilities and new experiences of networks, playing off the equivocation of the human experience of networks and the internal experience of more-than-human networks metaphorically represented by the slime mold.

In de Castro’s rendering, equivocation is not a product of the incommensurability of languages, but of perspectives that are located in different bodies; as such, it is a window to the outside, “not error, deception, or falsehood but the very foundation of the relation implicating it, which is always a relation with exteriority.” For de Castro “equivocation is not what prevents the relation, but what founds and impels it” (Castro & Skafish, 2014, p. 89), and, furthermore, “it is not a subjective weakness but a machine for objectification” (ibid.), where objectification does not mean reification or essentialisation, but rendering things objective. Or, in Ortega’s and Harman’s sense, recognising them as separate “I”s. De Castro remarks that equivocation is not a matter of symbolic incommensurability but the impossibility to exchange perspectives. The *Physarum* network projects described above explore the possibilities of exchanging perspectives between humans and slime mold, producing defamiliarized representations of human collectives and infrastructures, but more importantly, produce new perspectives and points of view—those of networks, which constitute the ultimate equivocal concept connecting humans and slime mold and traversing disciplines.

## Conclusions

Approaching *Physarum* network models as boundary objects allows to investigate how they mediate between different social domains, with their respective practices and concepts. All projects discussed above are interdisciplinary: the first one was a collaboration between biologists and computer programists, while Adamatzky’s and Grant’s combine computer science with art. In the network experiments the slime mold’s protoplasmic tubes operate like a black box, producing outputs from inputs without any necessity of understanding and exploring the internal mechanisms responsible for performing the task, which makes the practices of cultivating and exploring *Physarum* networks easily adaptable by new research milieus; their black box quality makes them open to a variety of research questions and guiding narratives. Each experiment includes the material work of abstracting network patterns from the slime mold’s body, which in each case is guided by different questions and stories; not just the networks but the material process of abstraction is the structure that takes different meanings in different social context and constitutes *Physarum* as a boundary object between them.

When travelling among disciplinary context the methods of comparison change too: what works as an analogy in network science can become a metaphor in an art project. The tension between the representative and non-representative function in the case of *Physarum* networks is the space of equivocation where creativity happens, representation is not assumed but achieved via abstraction and the metaphors that guide it. Representations are thus tools, but also artefacts produced in the process of translation between the slimy and the human: Grant’s projects engage in a playful exploration of the inaccuracy or failure of such translation. According to de Castro “To translate is to take up residence in the space of equivocation. Not for the purpose of cancelling it (that would suppose that it never really existed) but in order to valorize and activate it, to open and expand the space imagined not to exist between the (conceptual) languages in contact” (Castro & Skafish, 2014, p. 89). This effect turns *Physarum* into a productive boundary object, which does much more than just foster collaboration across social worlds. According to the anthropologist of science Joan Fujimura, boundary objects destabilize boundaries between disciplines by influencing their respective methodologies and reconstituting their objects of study (Fujimura, 1992). Such is the function of *Physarum*, which, while travelling between biology, network science and art, remakes the concept of network and the narrative frameworks that underlie the network modelling practices.

According to Munster networks experience themselves too, since they form more-than-human assemblages to which networked experience is internal. In Adamatzky’s and Grant’s projects networks are aesthetic objects to be experienced and affected by; the slime mold as a living network is a tool for exploring networks as experience not abstraction. By exploring the ungraspable inwardness of networks *Physarum* experiments effectively counteract the numbness that results from the ubiquity of networks and point to the relationality that is the foundation of network experience. The materiality of slime mold exposes network abstraction as a material process of producing relations—because it can be guided by a variety of narratives, metaphors, and ontological commitments, it opens up a space of indeterminacy and conceptual creativity in thinking about relations between slime mold and humans.

## Data Availability

Non-applicable.

## Footnotes

1 In the standard binomial nomenclature used in biology *Physarum* is the name of the genus and *polycephalum* the name of the species, the standardized notation being *P.polycephalum*. I use the name *Physarum* as it is used in this form by the computer scientists and artists whose works I am discussing in this paper.

2 US National Institutes of Health provide an inventory of thirteen model organisms for biomedical research, including mouse, rat, zebrafish, fruit fly, nematode, baker’s yeast, and thale cress (Ankeny & Leonelli, 2021, p. 2).

3 Audrey Dussutour, Center for Research on Animal Cognition, personal communication.

4 Artist Heather Barnett founded the online community SLIMOCO specifically for the purpose of facilitatiing sch exchanges among artists, amateur biologists and other slime mold afficionados.

5 The internal mechanisms of protoplasmic streaming and the complex oscillatory system in *Physarum* cell that is responsible for its movements have been described by a number of scholars (Boussard et al., 2021; Jones, 2015; Mayne et al., 2015). This is just to say that in network modelling experiments an understanding of those processes is not necessary.

6 See: https://phychip.eu/ (accessed: 16 April 2026).

7 The notion of territory as an empty space has a long genealogy retraced by Henri Lefebrve (Lefebvre, 1992) who has demonstrated that the fact that we can imagine social space as an abstract empty surface is a product of a long historical process of state formation and governance of territories—another practice of material abstraction.

8 See also the works of ecoLogic studio (Poletto & Pasquero, 2012), Theresa Schubert (Schubert et al., 2015) and Jenna Sutela (Sutela, 2017).

9 Sarah Grant, personal communication.

10 Sarah Grant, personal communication.

11 As part of the exhibition “Under the Viral Shadow”; see https://artlaboratory-berlin.org/exhibitions/under-the-viral-shadow (accessed: 16 April 2026).
